# Effect of Oral Nutritional Supplementation on Adequacy of Nutrient Intake among Picky-Eating Children at Nutritional Risk in India: A Randomized Double Blind Clinical Trial

**DOI:** 10.3390/nu15112528

**Published:** 2023-05-29

**Authors:** Fahmina Anwar, Menaka Yalawar, Pranali Suryawanshi, Apurba Ghosh, Pramod Jog, Anuradha Vaman Khadilkar, Bala Kishore, Anil Kumar Paruchuri, Prahalad D. Pote, Ravi D. Mandyam, Sandeep Shinde, Atish Shah

**Affiliations:** 1 Abbott Nutrition, Research & Development India, Mumbai 400051, India; 2 Biostatistics and Statistical Programming, Life Sciences-Digital Business Operations, Cognizant Technology Solutions India Private Limited, Bengaluru 560045, India; menaka.shekarappa@abbott.com; 3 Biostatistics and Statistical Programming, Life Sciences-Digital Business Operations, Cognizant Technology Solutions India Private Limited, Navi Mumbai 400708, India; pranali.suryawanshi@abbott.com; 4 Institute of Child Health, Kolkata 700017, India; apurbaghosh@yahoo.com; 5 Medipoint Hospital, Pune 411007, India; dr_pramodjog@yahoo.co.in; 6 Jehangir Clinical Development Centre, Jehangir Hospital, Pune 411001, India; anuradhavkhadilkar@gmail.com; 7 Saint Theresa’s Hospital, Hyderabad 500018, India; baki2004@gmail.com; 8 Praveen Cardiac Centre, Vijayawada 520010, India; paruchuriak70@gmail.com; 9 Noble Hospital Private Limited, Pune 411013, India; drppote@gmail.com; 10 JSS Hospital, Mysuru 570004, India; ravimdped@gmail.com; 11 Pune Sterling Multispecialty Hospital, Pune 411044, India; drsandeepshinde@gmail.com; 12 Sangini Hospital, Sangini Complex, Ahmedabad 380006, India; dr_atish78@yahoo.com

**Keywords:** picky-eating children, oral nutrition supplement, nutrient adequacy, dietary diversity, food intake adequacy, food consumption pattern, dietary counseling

## Abstract

Nutrient inadequacies among picky-eaters have adverse effects on growth and development. Oral nutritional supplements (ONS) along with dietary counseling (DC), rather than DC alone as reported in our earlier publication, promoted growth among picky-eating Indian children aged from >24 m to ≤48 m with weight-for-height percentiles lying between the 5th and 25th (based on WHO Growth Standards) over 90 days. This paper presents the contribution of ONS to nutrient adequacy, dietary diversity, and food consumption patterns in children (N = 321). Weight, height, and dietary intakes, using 24-h food recalls, were measured at baseline (Day 1) and at Days 7, 30, 60, and 90. Nutrient adequacy, dietary diversity score (DDS), and food intake adequacy were calculated in both the supplementation groups (ONS1 + DC and ONS2 + DC; n = 107 in each group) and the control group (DC-only; n = 107). Supplements increased nutrient adequacy in both of the ONS + DC groups relative to control (*p* < 0.05). The proportions of children with adequate nutrient intakes increased significantly at Day 90 in the supplemented groups as compared to in the control group (*p* < 0.05), especially for total fat, calcium, vitamin A, vitamin C, and thiamin. Although no significant differences were observed in DDS in any of the groups, the percentage of children consuming ≥4 food groups in a day had increased in all the groups. Consumption of fruit and vegetables and cereals had increased significantly from baseline to Day 90. ONS along with dietary counseling was found to have improved nutritional adequacy without interfering with the normal food consumption patterns of picky-eating children at nutritional risk.

## 1. Introduction

Globally, 149.2 million under-age-five children are stunted, 45.4 million children are wasted, and 38.9 million children are overweight or obese [1]. Data from the National Family Health Survey-5 (2019-21), the latest in its series [2], reported a slight decline in stunting rates (35.5%) when compared to the NFHS-4 data (38.4%) [3]. The prevalence of undernutrition remains high in under-age-five children living in the Indian subcontinent. Preschool years are important for formulating food habits, food likes and dislikes, and eating behaviors that are more firmly established during late childhood, adolescence, and young adulthood [4,5,6]. Feeding problems often place children at risk of malnutrition. Many feeding problems resolve with age; however, they may remain persistent in some situations during adolescence and adulthood [7,8,9,10].

Children with picky-eating behaviors often lack adequate nutrition in their diet, and this compromises their growth and development. The prevalence of picky-eating in children is observed to be highest during the age range of 2–5 years, when it ranges from 10 to 50% [7,10,11,12,13] in different published studies, globally. Picky-eating is defined variably in literature [12]; however, it includes aspects like lack of dietary diversity [14,15,16], the eating of inadequate amounts of food, strong food likes and dislikes [7,10], neophobia [10], and difficult mealtimes [17], causing major concerns for parents. Reduction in food consumption and low variety in diet predispose these children to various nutritional deficiencies [13,18,19], lower weights [7,13,20] and heights [7,20], and lower IQs [21]. Picky-eating is also found to be associated with lower intake of fruits and vegetables [11,13,14,19,22] and higher intake of palatable energy-dense foods [19,22,23,24] that may lead to excessive weight or obesity in children. Picky-eating behaviors not only compromise growth but also predispose children to infections and gastrointestinal diseases like constipation [25,26].

Multicomponent approaches have been beneficial in improving picky-eating behavior and nutritional status in children [27]. The combination of nutritional supplementation and dietary counselling acts as a tool in many programs for managing both the immediate and underlying factors of malnutrition [28,29]. Nutritional supplementation has been shown to have a beneficial impact on growth in children [26,30,31,32,33,34] and increase nutrient contribution in their diet [33,34,35,36,37,38] without altering their food consumption patterns.

The main goal of the present study is to compare the efficacy and safety of using two pediatric oral nutritional supplements (ONS) along with dietary counseling vs using dietary counseling alone on the growth of picky eaters who are at risk of malnutrition in India. Our earlier publication presented the effect of ONS and dietary counselling on growth in children [30]. This is a follow-up publication that presents the influence of ONS on nutritional adequacy and food consumption patterns, and their effects on the usual diets of children.

## 2. Materials and Methods

### 2.1. Study Design and Participants

This was a multi-centric prospective randomized double-blinded study with two parallel treatment groups and an open-labelled control group. It was conducted in order to examine the impact of oral nutritional supplementation on growth and nutrient intake in picky-eating children between 24 and 48 months of age. The study was conducted in 10 hospitals and clinics in different cities in India. The intervention lasted for a period of 90 days and the entire duration of the study was approximately 12 months (i.e., between June 2016 and February 2017). Data on anthropometry (weight, height, and mid-upper-arm circumference) and dietary intake were collected at five time points, viz., Days 1 (Baseline), 7, 30, 60, and 90. Appetite score, palatability, and compliance were also recorded for each subject during each visit.

Two types of supplements were given to the children: (1) ONS1, a milk-based supplement, and (2) ONS2, a lactose-free supplement (manufactured by PediaSure and PediaSure Advance, respectively, at Abbott Healthcare Private Limited, Mumbai, India). These contained three macronutrients with matching levels of protein (at 12% of energy), 28 vitamins and minerals, and the prebiotic fiber fructo-oligosaccharide (FOS). The fat energy percent was higher, and the carbohydrate energy percent was lower, in ONS2 than in ONS1 (Table 1). Both types of supplements had similar nutrient compositions [30].

A total of 321 children, who had weight-for-height was between the 3rd and 15th percentiles according to the current WHO Growth Charts (2006) [39] and were identified as picky eaters, were enrolled in the study. The detailed inclusion and exclusion criteria were the same as in our earlier published study [30]. The enrolled children were randomized in three groups (n = 107 in each group), which were administered the following treatments: (i) Oral Nutrient Supplement 1 along with Dietary Counselling (ONS1 + DC), (ii) Oral Nutrient Supplement 2 along with Dietary Counselling (ONS2 + DC), and (iii) Dietary Counselling only (DC-only), which was administered to the control group. The randomization was computer-generated using a pseudo-random permuted blocks algorithm. Randomization was stratified by the study center. Blinding was observed as none of the investigators or their staff, members of Abbott Nutrition’s staff, or families of the children were informed of the ONS identities of the subjects during the study period. At baseline, the parents of Group 1 and Group 2 were trained on the method of reconstitution, dosage, and storage of ONS 1 and 2, respectively. A measuring shaker (calibrated to 200 mL) was provided for the reconstitution of ONS powder and for measuring ONS consumption. The children in the ONS groups took a minimum of 1 serving/day, or a maximum of 2 servings/day, during the 90-day intervention interval.

All three groups received dietary counselling. Dietary counselling on the consumption of a balanced diet, the inclusion of a variety of foods in a daily diet, improving diet quality, and improving appetite for meeting the nutrition requirements of a child were given by a trained dietitian at each visit. Compliance was assessed via diary records kept by the parents/guardians; children were considered compliant if they had consumed at least 75% of the recommended ONS intake. Anthropometric measurements of weight, height, body mass index (BMI), and mid-upper-arm circumference (MUAC) were taken on Days 1, 30, 60, and 90 for all three groups by study staff who had been trained to use standardized methods. The data on changes in growth from Day 1 to Day 90 were assessed using various anthropometric indicators, and have been reported in our earlier publication [30]. This paper focuses on the changes in nutrient intake and adequacy in terms of Estimated Average Requirements (EARs), as recommended by the Indian Council of Medical Research–National Institute of Nutrition (ICMR–NIN) [40], from Day 1 to Day 90.

### 2.2. Dietary Assessment

Dietary intake was assessed using the 24-h recall method at each visit by a trained dietitian. In each 24-h dietary recall, the respondent was asked to remember and report all foods and beverages consumed in the preceding 24 h or in the preceding day, and the data was recorded using standard cups and measures. The interview conducted was structured, with specific probes, to help the respondent remember all the foods and beverages consumed throughout the day. Data on food and nutrient intake was analysed at each visit by the trained dietitian at the Madras Diabetes Research Foundation (MDRF), Chennai. Nutrient adequacy was assessed for nine nutrients (energy, protein, carbohydrate (CHO), total fat, iron, calcium, vitamin A, thiamine, and Vitamin C). The nutrient adequacy for each nutrient was computed as a percent of the age-specific requirement for that nutrient, i.e., using the Estimated Average Requirements (EARs) given by ICMR–NIN [40]. EARs have been given separately for the 1–3-year and 4–6-year age groups by ICMR–NIN [40].

The intakes of various food groups were computed using the 24-h recall method at each visit. Food intake adequacy was assessed for 8 food groups, viz., (1) milk and its products, (2) pulses, legumes and millets, (3) meat, fish, eggs, and poultry, (4) fruits and vegetables, (5) tubers, (6) cereals, (7) fats, nuts, and oilseeds, and (8) added sugars. Food intake adequacy for each food group was calculated as a percentage of the food intake required for a recommended balanced diet for children in the 1–3-year and 4–6-year groups as per ICMR–NIN [41]. Nutrient and food adequacies were dichotomized as adequate (≥100) and inadequate (<100).

Data was also used to calculate Dietary Diversity Scores at each visit. Individual Dietary Diversity is a qualitative measure of food consumption that can be used as a proxy for the nutrient adequacy of an individual’s diet. Dietary Diversity Scores have been validated for various age/gender groups as proxy measures for both the macronutrient as well as the micronutrient adequacies of diets [42]. In our study, food in the 24-h dietary recalls was categorized into 9 food groups: (1) Starchy staples, (2) dark green leafy vegetables, (3) vit-A-rich fruits and vegetables, (4) other fruits and vegetables, (5) organ meat, (6) meat and fish, (7) eggs, (8) legumes, nuts, and oilseeds, and (9) milk and milk products. If at least one food from a particular food group had been consumed, that group was scored 1. If no food from a particular food group had been consumed, that group was scored 0. The individual scores of each of the food groups were summed up to obtain a total score out of 9. Scores of 1–3 were considered representative of low dietary diversity, 4–7 of medium dietary diversity, and >7 of high dietary diversity. The parents of the children in experimental group 1 and experimental group 2 were given parental diaries that included records on the consumption of medications, fortified beverages other than the study product, and nutritional supplements received during each visit. All of these details needed to be updated on a weekly basis. The parental diaries also contained product intake records, which were filled in by the parents of the children in both experimental treatment study groups. These needed to be updated daily. Parental diaries were also provided for the control group in order to record the consumption of medications, fortified beverages, and nutritional supplements at Days 1, 7, 30, 60, and 90.

### 2.3. Ethics

The study was registered at clinicaltrials.gov in the US (Abbott Nutrition 2015, NCT02523027) [43] and with the Clinical Trials Registry-India (Abbott Nutrition International India 2015, CTRI/2015/10/006330) [44]. The study was performed in accordance with protocol, Good Clinical Practice (GCP) guidelines [45], regulations governing clinical study conduct, and the ethical principles that have their origin in the Declaration of Helsinki. The study protocol, all amendments, and the informed consent form were reviewed and approved by the Independent Ethics Committees/Institutional Review Boards of all 10 study hospitals. The parents or guardians of the children voluntarily gave written informed consent prior to enrollment.

### 2.4. Statistical Analyses

All randomized subjects who consumed any amount of the study product were included in the intention-to-treat (ITT) cohort, and all analyses were conducted on the ITT dataset. Continuous variables were analyzed at baseline and post-intervention using parametric tests (analysis of covariance (ANCOVA) and paired *t*-test) unless the distribution of any given variable was declared non-normal, in which case a non-parametric test (Wilcoxon rank-sum test or signed-rank test) was conducted on that variable [46]. Residuals from the parametric analysis were used to check for deviation from normality by using a combination of methods (stem-and-leaf plot, normality plot, and Shapiro-Wilk test). All hypothesis testing, except for the tests for interaction and normality, was done using 2-sided, 0.05-level tests of significance. The step-down Bonferroni (Holm) procedure was used to adjust the significance levels for multiple comparisons.

For analyses of baseline variables with ANCOVA, age, gender, and study site were used as covariates. For each nutrient, food intake and its adequacy and the changes-from-baseline in each treatment group were analyzed using a one-sample paired *t*-test, or a signed-rank test if the data was declared non-normal. Each categorical variable was analyzed using a chi-square test or Fisher’s exact test. Tests for interactions and normality were done using two-sided 0.10- and 0.001-level respectively. When an interaction was significant, the step-down Bonferroni (Holm) procedure was used to adjust the significance levels for multiple comparisons. Release 9.4 of the statistical software SAS (SAS Institute Inc., Cary, NC, USA) was used. A *p*-value < 0.05 was considered statistically significant.

## 3. Results

At baseline (Day 1), a total of 321 children were enrolled in the study, with 107 in each group. The median age was 2.93 years and 39.9% of subjects were girls. There were no significant differences in mean weight, height, MUAC, and BMI z-scores between children in different groups (Table 2) after adjusting for the effects of age at enrollment, site, and gender. The mean maternal age was 27.98 years and 64% of the subjects’ mothers were housewives whereas the mean age of the subjects’ fathers was 32.67 years and 97.8% of them were employed. Most of the subjects’ families were nuclear (70.7%). There were no statistically significant differences between the groups at baseline. The CONSORT flow diagram of the study was provided in our earlier publication [30].

### 3.1. Changes in Growth in Children

A significant increase was observed in weight and BMI over time in the two ONS groups compared to what was observed in the DC-only group (*p* < 0.05). Mid-upper-arm circumference significantly improved in the ONS1 + DC group as compared to the DC-only group. An increase in height was observed in both the supplementation groups as compared to the DC-only group from Day 1 to Day 90, although the difference was not statistically significant. No significant differences in anthropometric measurements were observed between the two ONS groups. The data on these measures was presented in our earlier publication [30].

### 3.2. Changes in Nutrient Intake of Children

The nutrient intake results are presented in Table 3. At Day 1, there was no significant difference in nutrient intakes among the three groups, except when it came to the intakes of Vitamin A (ONS1 + DC vs. DC-only and ONS2 + DC vs. DC-only; *p* < 0.001); however, at Day 90, the intakes of all nutrients were found to be significantly higher in both the supplementation groups (ONS1 + DC and ONS2 + DC) as compared to the control group (DC-only) over time (*p* < 0.05). No significant difference was observed between the two ONS groups. Mean total daily energy intake was significantly higher in all the groups at Day 90; however, the difference in energy intake was much higher in both the supplementation groups (+281.9 kcal in ONS1 + DC and +277.6 kcal in ONS2 + DC) as compared to the DC-only group (+130.6 kcal). Similarly, significantly higher intakes of protein, CHO, total fat, iron, calcium, Vitamin A, thiamin, and Vitamin C were also observed in children in all the three groups (*p* < 0.05). However, the increment in the intake of all these nutrients was much higher in both the supplemented groups as compared to the DC-only group.

### 3.3. Changes in Nutrient Adequacy in Children

**Figure 1:**
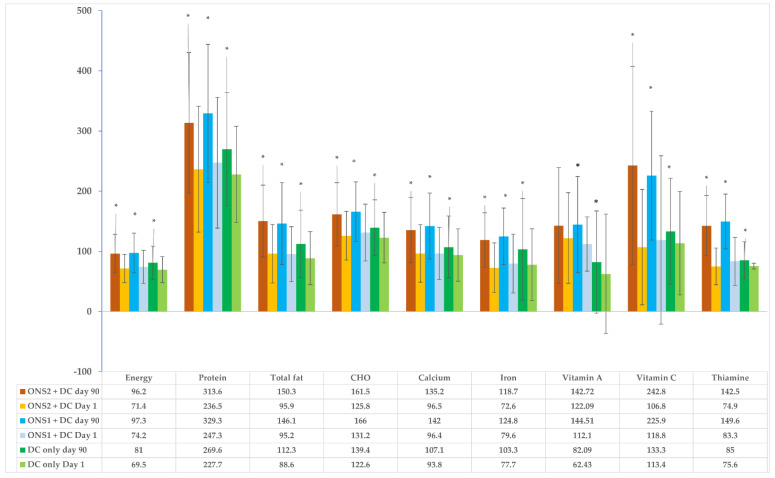
Mean nutrient adequacies of various nutrients for ONS1 + DC, ONS2 + DC and DC-only groups at Day 1 and Day 90 in children. (Error bars indicate SD values and *, Significant difference in mean nutrient adequacy between day 1 and day 90 at *p* < 0.05).

Nutrient adequacy was computed to assess the changes in all the three groups over time. The mean (SD) values of the energy intakes of the children at baseline were 880.87 (299.89) kcal in the ONS1 + DC group, 853.12 (249.70) kcal in the ONS2 + DC group, and 825.46 (259.45) kcal in the DC-only group, indicating that they were consuming nearly 70% of the recommended Estimated Average Requirements (EARs) for their ages and genders (note: the EAR for energy is equivalent to the estimated energy requirement (EER)). The children had adequate intakes of protein, carbohydrates, and vitamin C at baseline. The intakes of total fat and calcium were slightly lower than the EAR values, whereas the intakes of iron, vitamin A (in the DC-only group), and thiamin were much below the recommended nutrient levels (Figure 1).

**Figure 2:**
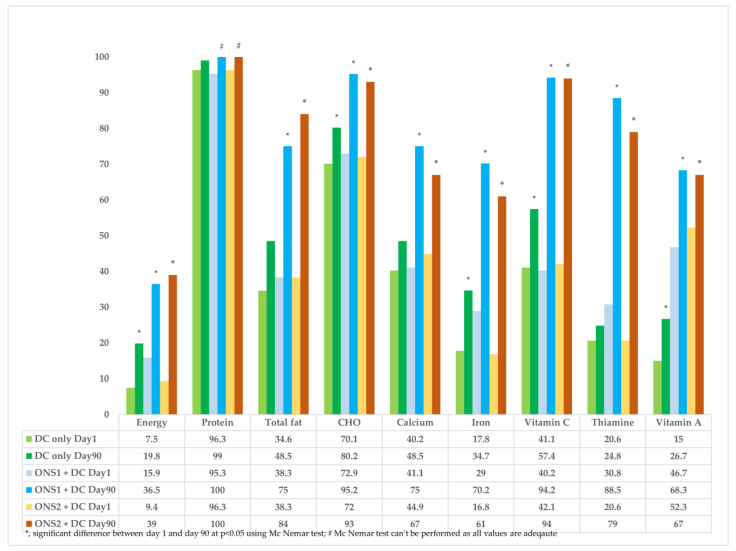
Percentages of children with adequate intake of various nutrients for the ONS1 + DC, ONS2 + DC, and DC-only groups at Day 1 and Day 90.

The mean daily energy intakes of children in both the supplementation groups were found to have significantly increased at Day 90 from their values on Day 1 (*p* < 0.05), and had reached more than 95% of the EARs in both the groups as compared to 81% in the DC-only group (Figure 1). It was observed that only 15.9% children in the ONS1 + DC group, 9.4% in the ONS2 + DC group, and 7.5% in the DC-only group had had adequate energy intakes on Day 1. The percentage of children with adequate energy increased significantly in all the three groups (*p* < 0.05). More than 95% of the children had had adequate intake of protein at baseline, and this value reached 100% at Day 90 in both the supplementation groups and 99% in the DC-only group (Figure 2).

The intake of total fat, which was marginally lower than the EAR at baseline, reached adequacy at Day 90 for all the three groups. Only one-third of the children were consuming adequate fat in their diets at Day 1. Carbohydrate and vitamin C intake was adequate in all the three groups at Day 1 and increased at Day 90, though the difference in intake was significantly higher among the supplemented groups as compared to the control group (Figure 1). The percentage of children consuming adequate fats increased significantly in both the supplemented groups (75% in ONS 1 + DC and 84% in ONS2 + DC; *p* < 0.001) at Day 90; however, the increase was not significant in the DC-only group at Day 90 (48.51%, *p* = 0.058).

Seventy percent of the children were consuming carbohydrates adequately in their diet at baseline; this value was found to have increased significantly in all the three groups (ONS1 + DC—95.2%, ONS2 + DC—93%, and DC-only—80.2%, *p* < 0.05) at Day 90. The number of children consuming vitamin C adequately increased significantly from 40% at Day 1 to more than 90% at Day 90 in both the supplemented groups (*p* < 0.001) as compared to only a 16.3% increase in the DC-only group (Day 1—41.1%, Day 90—57.4%; *p* = 0.005).

The intakes of iron increased significantly and reached more than 100% of the EAR in all the three groups (Figure 1). However, the increase in the number of children consuming adequate amounts of iron from Day 1 to Day 90 was much higher in both the supplemented groups (ONS1 + DC—70.2%, ONS2 + DC—61%; *p* < 0.001) as compared to the DC-only group (34.7%, *p* = 0.004).

In the children, calcium intake was more than 90% of the EAR and thiamin intake was between 70–80% of the EAR at Day 1. Both values increased significantly to more than 100% in all the groups at Day 90, except for the value of thiamin in the DC-only group (Day 90—85%). The proportion of children consuming adequate calcium and thiamin increased significantly in both the ONS1 + DC group and the ONS2 + DC group (*p* < 0.05) as compared to the DC-only group (calcium: Day 1—40.2%, Day 90—48.5%, *p* = 0.286; thiamin: Day 1—20.6%, Day 90—24.8%, *p* = 0.602), as shown in Figure 2. A detailed table is presented as part of the Supplementary Materials (Table S1).

Vitamin A intake was adequate in both ONS1 and ONS 2 groups at baseline and day 90, and significantly higher than the DC-only group at both time points, with the baseline differences among the groups persisting to Day 90 (Figure 1). The percentages of children consuming adequate Vitamin A were found to have increased significantly in all the three groups at Day 90; however, the number of children consuming Vitamin A adequately was much higher in both the supplemented groups at baseline and Day 90 (Figure 2).

Overall, the findings indicated that children in both the supplemented groups met their nutrient requirements (EARs) at Day 90 as compared to the children in the DC-only group. There were no differences in the nutrient adequacies of various nutrients between the two supplemented groups. Post-intervention, significantly higher percentages of children were consuming various nutrients adequately in both the ONS1 + DC group and ONS 2 + DC group relative to the DC-only group. The relevant data is presented in the Supplementary Materials (Table S1).

Appetite scores improved significantly in both the supplemented groups from Day 1 to Day 90 compared to the control group (ONS1 + DC-*p* = 0.006, ONS2 + DC-*p* = 0.024). The palatability of each of the two supplements was marked on the Hedonic Faces scale by the parent/LG on each visit. It was observed that there were no significant changes in the palatability of either supplement at Day 90.

### 3.4. Changes in Food Consumption Pattern and Dietary Diversity over Time

**Figure 3:**
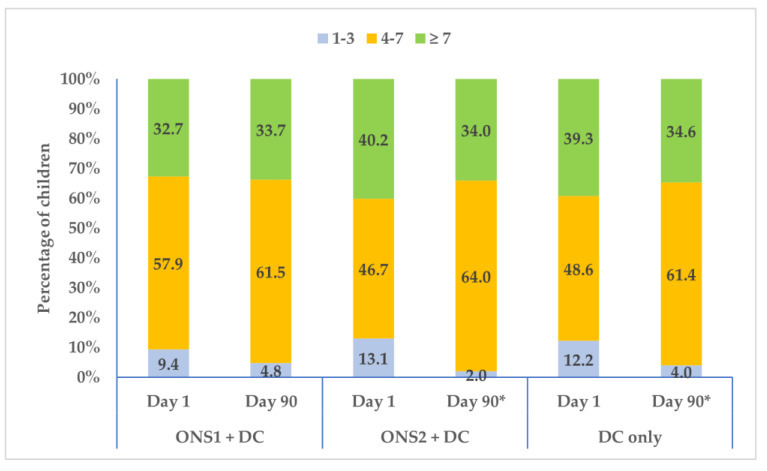
Percentage of children with Dietary Diversity Scores for ONS1 + DC, ONS2 + DC and DC-only groups at Day 1 and Day 90. *, significant difference between Day 1 and Day 90 at *p* < 0.05.

Mean dietary diversity in children did not significantly change at Day 90 from baseline except in Group 1 (ONS1 + DC: Day 1—5.90 ± 1.94, Day 90—6.15 ± 1.69, *p* = 0.048; ONS2 + DC: Day 1—6.02 ± 2.10, Day 90—6.11 ± 1.61, *p* = 0.166; DC-only: Day 1—5.94 ± 1.90, Day 90—5.95 ± 1.63, *p* = 0.620). The percentages of children consuming ≥ 4 food groups increased in all the three groups, though it did so significantly (*p* < 0.05) in the ONS2 + DC and DC-only groups (Figure 3), suggesting that continuous dietary counselling may improve dietary diversity.

Table 4 presents the mean consumptions of various food groups by the children in the three groups at Day 1 and Day 90. There was no difference in the consumptions of all the food groups at Day 1 between the supplemented groups and the control group. While changes in the consumption of some food groups differed between Day 1 and Day 90 within groups, these changes among groups were insignificant for all the food groups at Day 90 as well, suggesting that food consumption patterns did not vary with supplementation. There were no significant differences in the consumptions of various food groups between the two supplemented groups. The intake of fats, nuts, and oilseeds increased significantly from Day 1 to Day 90 in both the supplementation groups, as these nutrients are supplied by the supplements (ONS1 + DC-*p* = 0.007, ONS2 + DC-*p* = <0.001). It was also observed that the intakes of pulses (*p* = 0.013), meat, fish, and poultry (*p* = 0.017), fruits and vegetables (*p* = 0.001), cereals (*p* = 0.009), and fat (*p* < 0.001) also increased significantly in the DC-only group from Day 1 to Day 90. Likewise, the intakes of fruits and vegetables (ONS1 + DC-*p*= 0.024, ONS2 + DC-*p* = 0.021) increased significantly in the supplementation groups. Regular dietary counselling had had a positive impact on the food consumption patterns of the children in the study.

**Figure 4:**
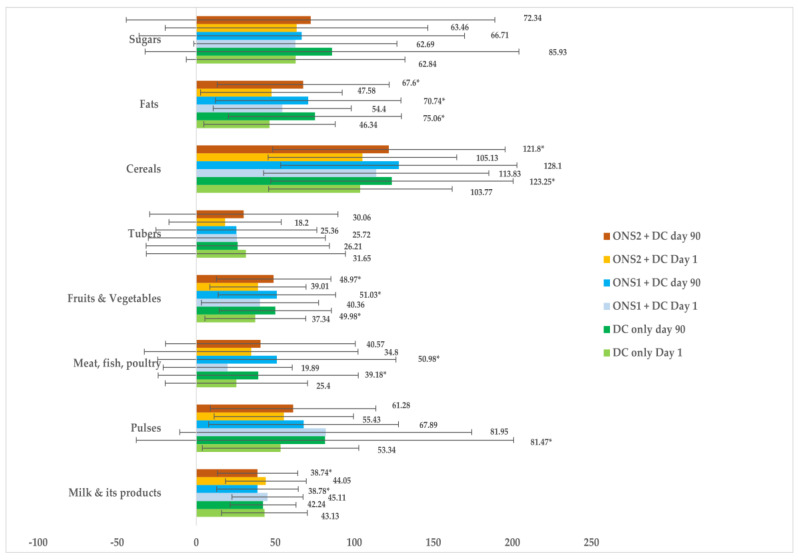
Mean percent adequacy for the consumption of various food groups compared with the recommendations for a balanced diet in children by NIN−ICMR, 2011. (Error bars indicate SD values and * indicates a significant difference in mean nutrient adequacy between day 1 and day 90 at *p* < 0.05).

The consumption of cereals was adequate in all the three groups at baseline and Day 90, when compared with the recommendations for a balanced diet in young children by ICMR−NIN [41] (Figure 4). Adequate consumption of meat, fish, egg, and poultry increased significantly from Day 1 (19.89%) to Day 90 (50.98%) in the ONS1 + DC group (*p* < 0.001). The consumption of adequate amounts of fruits and vegetables increased significantly from 40% to nearly 50% in both the supplemented groups (ONS1 + DC-*p* = 0.010; ONS2 + DC-*p* = 0.029) and the DC-only group (*p* = 0.005). A significant increase was observed in the consumptions of adequate amounts of pulses and legumes (*p* = 0.013), meat, fish, egg, and poultry (*p* = 0.017), cereals (*p* = 0.005), and fats (*p* < 0.001) in the DC-only group from baseline at Day 90 (Figure 4). The percentages of children consuming adequate amounts of various food groups did not change significantly in any of the groups from Day 1 to Day 90, except when it came to the consumption of adequate amounts of fats and oils and meat, fish, egg, and poultry in the ONS1 + DC and DC-only groups (*p* < 0.05). The relevant data is presented in the Supplementary Materials (Table S1).

## 4. Discussion

Picky-eating children are at risk of malnutrition, and nutrition intervention may play an important part in achieving the optimal growth trajectory in these children. Malnutrition in children is a common underlying reason for increased susceptibility to respiratory and gastrointestinal infections and increased risk of mortality [28,47]. There is strong evidence to suggest that protein energy malnutrition and deficiencies of various micronutrients—especially iron, iodine, and vitamin A—early in life lead to poor growth and development [28,48,49]. Timely and effective interventions are key to promote catch-up growth in children at the risk of malnutrition. Moreover, if children are picky-eaters, their food intake is affected, making them prone to nutritional deficiencies and compromised growth. A picky-eater consumes less calories as compared to a non-picky eater and is more likely to have lower weight-for-age [7,13,22,50,51,52] and lower height-for-age [7,22]. The children in this study had been consuming 70% of the recommended energy intake. Their intakes of total fat, calcium, iron, vitamin A, and thiamin were much below the age−appropriate EARs. A study on 151 picky-eating children in China aged 2.5–5 years reported similar intake deficits that is, the subjects’ intakes of energy were 75%, and intakes of calcium, iron, zinc, and vitamins C and E ranged from 52–73%, of the Recommended Nutrient Intakes (RNIs) [53]. Another study on Chinese children reported lower intakes of dietary protein, iron, fiber, and zinc [13]. However, a study on Danish children showed no significant differences in energy intake and diet quality between picky and non-picky-eaters [54].

Studies have shown that those adults who were picky-eaters in their childhood had had lower intakes of micronutrient-rich foods, such as fruit, vegetables, and whole grains [8,13,14,50,53,55], and more intake of energy-dense foods, such as snack foods and sugar-sweetened beverages [55,56], leading to poor health outcomes later in life [8]. A study conducted on 506 American preschoolers reported an inverse relationship between picky-eating behavior and the Healthy Eating Index Scores for whole fruit, total vegetables, greens and beans, and total protein foods [57].

Establishing healthy eating behaviors and optimizing nutritional requirements during preschool years are imperative to prevent malnutrition and other diet-related diseases, especially obesity and non-communicable diseases, in adult life. Nutritional supplementation has been shown to improve the nutritional statuses and cognitive abilities of undernourished preschool children [21,48]. Our study showed improvements in the weight-for-height and weight-for-age percentiles of preschool children in India after a 90-day intervention in both the supplemented groups [30]. Similarly, other studies conducted on picky-eating children in the Philippines [26,34], Taiwan [26], China [33], and Haiti [58] have also indicated improvements in the weight and height statuses of preschoolers after they received nutrient supplements.

In our study, ONS supplementation improved nutrient adequacy among picky-eating children as compared to what was found in the control or placebo group, especially the intakes of energy [33,59], carbohydrates [35], protein [59] and other micronutrients [33,59]. Dietary counseling along with nutritional supplementation improved the variety in diets, and the percent of children in our study who consumed more than 4 food groups in a day increased significantly post-intervention. Additionally, the consumption of fruits and vegetables, grains, and meat, fish, and poultry increased in all the three groups. Our results are consistent with previous studies that reported higher consumptions of fruits and vegetables [25,51,56], meat, and grains after the regular dietary counseling of picky eaters.

Nutritional support is essential for children recovering from illness. There are well-established studies indicating the relationship between malnutrition and infections [60]. Studies on picky-eating children have reported lower infection rates or early recoveries from infections among children who received nutritional supplements as compared to children in control groups [26]. A study conducted on Indian children showed less weight reduction and early recovery in children who were fed ONS as compared to children in the control group [32].

Multicomponent strategies may prove beneficial in improving nutrient intake and dietary pattern in children. The study clearly indicated that the nutrient intakes of the subjects were much lower than what had been recommended, and that supplements did not act as substitutes for other foods in the daily diets of children in the included age group. Thus, the additional intake of nutrients in the form of ONS could help children bridge the gap between their usual intakes and the recommended values without altering their usual food patterns until they start eating a variety of foods in adequate amounts in their daily diets. This practice will help children at mild risk of malnutrition catch up faster and also prevent further deterioration in their growth.

The strengths of this study are in its robust design, the completeness of dietary data in follow-up visits, the high rate of compliance, and a low loss of subjects in follow-up data. As a limitation, the subjects enrolled in the study were already at risk of undernutrition, and therefore, the study could not capture the effect of ONS on the other end of the spectrum. The findings of this study may not be generalized for other countries; however, they are of significance in countries like India where high wasting rates were observed in children who live in resource-poor settings [2] consuming poor quality diets and are predisposed to infections.

## 5. Conclusions

Children who received oral nutritional supplements with dietary counseling, when compared with those who received dietary counseling alone, showed greater increases in energy, iron, thiamin, Vitamin C, and calcium intakes, and a higher percentage of children from the former category met their respective EARs in our study. There were also significant improvements in appetite reported for the two supplementation groups versus for the control group over the 90-day period. Both supplements were readily accepted by the children, with a compliance rate of 99%, suggesting that these children, on consuming ONS (one serving contained 210 additional kcal, 6 g of protein, and other vitamins and minerals), met their nutrient requirements for optimal growth and development as recommended by ICMR–NIN [40] without altering their daily food consumption patterns. The study also emphasized that regular dietary counseling had beneficial effects on improving variety in daily diets, especially when it came to the consumption of fruits and vegetables and grains. The study supports multiple intervention strategies for improving nutritional status in picky-eating children. Nutritional supplementation, along with dietary counseling, is an effective way to promote better nutritional outcomes.

## Figures and Tables

**Table 1:** Nutrient composition of ONS1 and ONS2 used in the study for intervention.

Nutrients	ONS1		ONS2	
	Per 100 g	Per Serving (45.5 g)	Per 100 g	Per Serving (45.5 g)
Energy (Kcal)	462	210.21	468	212.94
Protein (g)	14.1	6.42	14.1	6.42
Total Fat (g)	17.0	7.74	23.4	10.65
Carbohydrate (g)	62.74	28.54	50.21	22.85
Vitamin C (mg)	44	20.02	44	20.02
Vitamin A (mcg)	305	138.78	305	138.78
Thiamin (mg)	0.9	0.41	0.9	0.41
Iron (mg)	5.5	2.50	5.5	2.50
Calcium (mg)	386	175.63	386	175.63

**Table 2:** Socioeconomic and anthropometric statuses of children at enrollment (Day 1).

	ONS1 + DC (n = 107)	ONS2 + DC (n = 107)	DC-Only (n = 107)	Total (n = 321)	*p*-Value		
					ONS1 + DC vs. DC-Only	ONS2 + DC vs. DC-Only	ONS1 + DC vs. ONS2 + DC-Only
**Age in years ^a^** Median (Q1, Q3)							
1–3 years (n = 170)	2.53 (2.29, 2.74)	2.47 (2.18, 2.79)	2.51 (2.27, 2.72)	2.49 (2.20, 2.74)	1.000	1.000	1.000
4–6 years (n = 151)	3.41 (3.19, 3.67)	3.34 (3.17, 3.66)	3.40 (3.20, 3.74)	3.38 (3.18, 3.71)	1.000	1.000	1.000
Total (n = 321)	2.94 (2.46, 3.38)	3.01 (2.47, 3.35)	2.92 (2.47, 3.37)	2.93 (2.47, 3.35)	1.000	1.000	1.000
**Gender ^b^, n (%) (n = 321)**					0.485	0.678	0.648
Male (n = 193)	70 (65.4)	63 (58.9)	60 (56.1)	193 (60.1)			
Female (n = 128)	37 (34.6)	44 (41.1)	47 (43.9)	128 (39.9)			
**Family type ^b^, n (%) (n = 321)**					0.769	0.519	0.569
Nuclear (n = 227)	74 (69.2)	81 (75.7)	72 (67.3)	227 (70.7)			
Joint (n = 94)	33 (30.8)	26 (24.3)	35 (32.7)	94 (29.3)			
**Maternal age (years) ^c^** Mean (SD), (n = 321)	27.85 (4.04)	28.30 (3.42)	27.78 (3.60)	27.98 (3.69)	0.880	0.878	0.878
**Maternal employment ^b^, n (%) (n = 321)**					1.000	1.000	1.000
Employed Full time	16 (15.0)	19 (17.8)	14 (13.1)	49 (15.3)			
Employed Part time	2 (1.9)	2 (1.9)	2 (1.9)	6 (1.9)			
Unemployed	20 (18.7)	20 (18.7)	21 (19.6)	61 (19.0)			
Voluntary not working	69 (64.5)	66 (61.7)	70 (65.4)	205 (63.9)			
**Paternal age (years) ^c^** Mean (SD), (n = 320)	32.57 (4.23)	33.13 (4.56)	32.32 (3.74)	32.67 (4.19)	0.645	0.414	0.610
**Paternal employment ^b^, n (%) (n = 321)**					0.669	1.000	1.000
Employed Full time	106 (99.1)	103 (96.3)	105 (98.1)	314 (97.8)			
Employed Part time	0 (0.0)	1 (0.9)	2 (1.9)	3 (0.9)			
Unemployed	1 (0.9)	1 (0.9)	0 (0.0)	2(0.6)			
**Anthropometry ^d^** Mean (SD)							
Weight (kg) (n = 321)	11.18 (1.51)	11.12 (1.58)	11.04 (1.57)	11.11 (1.55)	1.000	1.000	1.000
Height (cm) (n = 321)	88.89 (6.97)	89.08 (7.19)	88.38 (7.15)	88.78 (7.09)	1.000	0.935	1.000
BMI (kg/m^2^) (n = 321)	14.11 (0.48)	13.98 (0.57)	14.10 (0.67)	14.06 (0.58)	0.986	0.055	0.090
MUAC (cm) (n = 321)	14.11 (1.47)	14.00 (1.39)	14.11 (1.37)	14.08 (1.41)	0.847	0.787	0.847

^a^ *p*-values are from Wilcoxon rank-sum tests. ^b^ Gender, family type, maternal and paternal employment of children were analysed using chi-square tests. ^c^ *p*-values are from the analysis of variance with treatment and site as factors. ^d^ *p*-values are from the analysis of covariance after adjusting for age, site, gender, and treatment with gender interaction.

**Table 3:** Mean/median intakes of various nutrients by the children in the supplementation and control groups at Day 1 and Day 90.

	ONS1 + DC				ONS2 + DC				DC-Only			*p*-Value for Day 1 *				*p*-Value for Day 90 *		
Nutrient	Day 1 (n = 107)	Day 90 (n = 104)	Mean/Median Difference	*p*-Value	Day 1 (n = 107)	Day 90 (n = 100)	Mean/Median Difference	*p*-Value	Day 1 (n = 107)	Day 90 (n = 101)	Mean/Median Difference	*p*-Value	ONS1 + DC vs. DC-Only	ONS2 + DC vs. DC-Only	ONS1 + DC vs. ONS2 + DC-Only	ONS1 + DC vs. DC-Only	ONS2 + DC vs. DC-Only	ONS1 + DC vs. ONS2 + DC-Only
Energy (Kcal)	880.87 (299.89)	1163.02 (366.33)	281.9 (422.2)	<0.001	853.12 (249.70)	1149.36 (357.09)	277.59 (70.64, 469.34)	<0.001	825.46 (259.45)	960.39 (309.30)	130.6 (336.7)	<0.001	0.475	0.895	0.895	<0.001	<0.001	0.916
Protein (g)	26.14 (10.08)	35.34 (11.50)	7.89 (−0.16, 15.61)	<0.001	25.02 (9.00)	33.36 (10.72)	8.81 (10.84)	<0.001	24.12 (8.16)	28.59 (9.81)	4.39 (9.60)	<0.001	0.524	0.866	0.866	<0.001	0.003	0.199
CHO (g)	131.15 (47.08)	166.0 (49.57)	34.3 (61.19)	<0.001	125.79 (40.48)	161.51 (52.59)	32.87 (−1.34, 61.00)	<0.001	122.61 (42.02)	139.35 (46.19)	16.32 (51.19)	0.002	0.601	0.769	0.769	<0.001	0.004	0.419
Total Fat (g)	23.79 (11.37)	36.52 (17.09)	12.63 (16.59)	<0.001	23.96 (12.21)	37.57 (14.88)	13.51 (15.91)	<0.001	22.16 (11.05)	28.07 (14.02)	5.68 (14.32)	<0.001	0.772	0.772	0.872	<0.001	<0.001	0.281
Iron (mg)	5.39 (3.14)	8.56 (3.05)	3.18 (3.76)	<0.001	5.01 (2.91)	8.16 (3.01)	2.91 (1.01, 5.44)	<0.001	5.42 (4.70)	6.99 (5.31)	0.65 (−0.39, 2.60)	<0.001	1.000	1.000	1.000	<0.001	<0.001	0.237
Calcium (mg)	407.51 (185.31)	603.08 (236.86)	195.3 (247.8)	<0.001	406.47 (193.89)	571.10 (223.17)	168.6 (232.0)	<0.001	395.74 (184.98)	452.72 (219.93)	52.31 (237.8)	0.029	1.000	1.000	1.000	<0.001	<0.001	0.419
Vitamin A (mcg)	229.14 (93.47)	295.59 (155.96)	24.11 (−8.10, 125.47)	<0.001	249.2 (135.19)	297.17 (217.57)	7.66 (−26.47, 87.26)	0.067	128.84 (215.09)	168.71 (177.16)	20.25 (−10.28, 88.97)	<0.001	<0.001	<0.001	0.377	<0.001	<0.001	0.233
Thiamin (mg)	0.57 (0.25)	1.03 (0.29)	0.46 (0.34)	<0.001	0.51 (0.19)	0.97 (0.30)	0.46 (0.34)	<0.001	0.52 (0.21)	0.58 (0.20)	0.06 (0.24)	0.016	0.489	0.942	0.489	<0.001	<0.001	0.058
Vitamin C (mg)	28.29 (31.76)	54.40 (24.58)	27.09 (16.19, 45.00)	<0.001	25.65 (21.54)	58.98 (39.83)	23.64 (12.09, 44.09)	<0.001	27.68 (22.14)	32.25 (21.07)	3.66 (−6.41, 17.89)	0.013	0.643	0.773	0.643	<0.001	<0.001	0.839

Data are presented in Mean (SD) form unless otherwise specified. Where data followed normality, the Mean (SD) of the difference is presented with *p*-values from the paired *t*-test, and otherwise, with *p*-values from the signed-rank test with the Median (25th and 75th percentiles) between Day 1 and Day 90 in each group. * *p*-values are from the Wilcoxon rank-sum tests for comparison between the three groups at Day 1 and Day 90.

**Table 4:** Mean consumption of various food groups for the ONS1 + DC, ONS2 + DC, and DC-only groups at Day 1 and Day 90.

	ONS1 + DC				ONS2 + DC				DC-Only				*p*-Value for Day 1 *			*p*-Value for Day 90 *		
Food Groups	Day 1 (n = 107)	Day 90 (n = 104)	Mean/Median Difference	*p*-Value	Day 1 (n = 107)	Day 90 (n = 100)	Mean/Median Difference	*p*-Value	Day 1 (n = 107)	Day 90 (n = 101)	Mean/Median Difference	*p*-Value	ONS1 + DC vs. DC-Only	ONS2 + DC vs. DC-Only	ONS1 + DC vs. ONS2 + DC-Only	ONS1 + DC vs. DC-Only	ONS2 + DC vs. DC-Only	ONS1 + DC vs. ONS2 + DC-Only
Milk and its products	225.57 (112.26)	193.90 (128.97)	−32.08 (140.1)	0.021	220.27 (128.19)	193.69 (127.13)	−26.94 (132.1)	0.044	215.64 (135.73)	211.19 (103.98)	−6.69 (118.9)	0.573	0.899	1.000	1.000	0.502	0.502	0.962
Pulses	24.58 (27.70)	20.37 (18.00)	−0.06 (−16.31, 10.76)	0.528	16.63 (13.23)	18.38 (15.71)	2.60 (19.24)	0.179	16.00 (14.83)	24.44 (35.79)	4.92 (−7.02, 15.70)	0.013	0.088	0.442	0.348	1.000	1.000	1.000
Meat, fish, poultry	9.95 (20.44)	25.49 (37.68)	0.00 (0.00, 30.89)	<0.001	17.40 (33.79)	20.29 (29.99)	0.00 (0.00, 16.72)	0.088	12.70 (22.54)	19.59 (31.66)	0.00 (0.00, 16.67)	0.017	1.000	1.000	1.000	0.860	0.969	0.969
Fruits and Vegetables	88.86 (80.06)	111.5 (77.21)	21.32 (94.67)	0.024	86.78 (66.64)	110.23 (83.36)	22.44 (95.32)	0.021	84.16 (74.99)	110.83 (77.67)	27.70 (−20.52, 69.55)	0.001	1.000	1.000	1.000	1.000	1.000	1.000
Tubers	15.68 (31.85)	18.03 (37.42)	0.00 (0.00, 2.61)	0.819	13.84 (26.75)	19.03 (32.61)	0.00 (0.00, 14.11)	0.169	18.98 (35.37)	17.51 (34.51)	0.00 (0.00, 11.35)	0.734	1.000	1.000	1.000	1.000	1.000	0.917
Cereals	90.11 (46.28)	100.82 (42.90)	10.42 (56.99)	0.065	84.25 (38.00)	96.30 (47.81)	7.17 (−21.33, 39.83)	0.053	82.23 (39.25)	95.30 (46.25)	13.15 (49.48)	0.009	0.750	0.982	0.982	0.667	0.905	0.667
Fats	13.60 (10.93)	17.68 (14.64)	4.00 (14.84)	0.007	11.89 (11.20)	16.9 (13.60)	3.43 (−2.56, 11.67)	<0.001	11.59 (10.39)	18.76 (13.68)	6.81 (14.61)	<0.001	0.208	0.923	0.208	0.787	0.741	0.787
Sugars	10.62 (10.73)	11.06 (15.98)	−1.58 (−5.33, 3.53)	0.247	10.79 (13.92)	12.55 (21.58)	0.00 (−5.05, 3.72)	0.602	10.83 (12.10)	14.50 (18.76)	0.00 (−3.66, 7.32)	0.231	1.000	1.000	1.000	0.215	0.292	0.850

Data are presented in Mean (SD) form unless otherwise specified. Where data followed normality, the Mean (SD) of the difference is presented with *p*-values from the paired *t*-test, and otherwise, with *p*-values from the signed-rank test with Median (25th and 75th percentiles) between Day 1 and Day 90 in each group. * *p*-values are from the Wilcoxon rank-sum tests for comparison between the groups at Day 1 and Day 90.

## Data Availability

Ethical restrictions imposed by the IRB prevent public sharing of the data for this study on children. The data used in this publication is owned by Abbott Nutrition. Data access requests will be evaluated by Abbott Nutrition in consideration of IRB requirements. Interested researchers will need to sign a research collaboration agreement with Abbott. Requests can be sent to fahmina.anwar@abbott.com.

## Supplementary Materials

The following supporting information can be downloaded at: https://www.mdpi.com/article/10.3390/nu15112528/s1, Table S1: Percentage of children consuming various nutrients and food groups in adequate amount in the three groups at Day 1 and Day 90.
