# Supplementary material for: Effect of Oral Nutritional Supplementation on Adequacy of Nutrient Intake among Picky-Eating Children at Nutritional Risk in India: A Randomized Double Blind Clinical Trial
Source: Nutrients. 2023 May 29;15(11):2528. doi: 10.3390/nu15112528 (PMC10255389; doi:10.3390/nu15112528)
Supplement: Supplementary file 1 [file nutrients-15-02528-s001.zip › nutrients-2381721-supplementary.pdf]

Supplementary Table S1: Percentage of children consuming various nutrients and food groups in adequate amount in the three groups at Day1 and Day 90 when compared with EAR and recommendations for balanced diet by ICMR-NIN

	**ONS 1 + DC**			**ONS2 + DC**			**DC only**		
	**Day 1 (n=107)**	**Day 90 (n=104)**	***p*-value**	**Day 1 (n=107)**	**Day 90 (n=100)**	***p*-value**	**Day 1 (n=107)**	**Day 90 (n=101)**	***p*-value**
Energy (Kcal)	17 (15.89 %)	38 (36.54%)	<.001	10 (9.35%)	39 (39.00%)	<.001	8(7.48 %)	20 (19.80 %)	0.014
Protein (g)	102 (95.33%)	104 (100)	NA	103 (96.26%)	100 (100.00%)	NA	103 (96.26%)	100 (99.01%)	0.180
Total fat (g)	41 (38.32%)	78 (75.00%)	<.001	41(38.32%)	84 (84.00%)	<.001	37 (34.58%)	49 (48.51%)	0.058
CHO (g)	78 (72.90%)	99 (95.19 %)	<.001	77 (71.96%)	93 (93.00%)	<.001	75 (70.09%)	81 (80.20 %)	0.034
Calcium (mg)	44 (41.12%)	78 (75.00 %)	<.001	48 (44.86%)	67 (67.00%)	<.001	43 (40.19%)	49 (48.51%)	0.286
Vitamin A (mcg)	50(46.73%)	71 (68.27%)	0.001	56 (52.34%)	67 (67 .00%)	0.013	16 (14.95%)	27 (26.73%)	0.048
Iron (mg)	31 (28.97%)	73 (70.19%)	<.001	18(16.82%)	61 (61.00%)	<.001	19 (17.76%)	35 (34.65%)	0.004
Vitamin C (mg)	43 (40.19%)	98 (94.23%)	<.001	45 (42.06 %)	94 (94.00%)	<.001	44 (41.12%)	58 (57.43 %)	0.005
Thiamine (mg)	33 (30.84%)	92 (88.46%)	<.001	22 (20.56 %)	79 (79.00%)	<.001	22 (20.56%)	25 (24.75%)	0.602
Milk & its products (g)	2 (1.87 %)	2 (1.92%)	1.000	3 (2.80%)	3 (3.00%)	0.564	4 (3.74%)	0	NA
Pulses (g)	30 (28.04%)	23 (22.12%)	0.274	16 (14.95%)	19 (19.00%)	0.178	13 (12.15 %)	21 (20.79%)	0.144
meat, fish, poultry (g)	13 (12.15%)	26 (25.00%)	0.004	19 (17.76 %)	22 (22.00 %)	0.317	13 (12.15%)	21 (20.79 %)	0.041
Fruits & Vegetables (g)	9 (8.41 %)	11 (10.58 %)	0.617	6 (5.61 %)	10 (10.00%)	0.248	5 (4.67%)	10 (9.90%)	0.132
Tubers (g)	10 (9.35 %)	10 (9.62%)	1.000	7 (6.54%)	5 (5.00%)	0.763	13 (12.15 %)	8 (7.92%)	0.439
Cereals (g)	56 (52.34%)	55 (52.88 %)	0.862	49 (45.79%)	52 (52.00%)	0.178	45 (42.06 %)	49 (48.51%)	0.257
Fats (g)	12 (11.21%)	25 (24.04%)	0.009	14 (13.08%)	20 (20.00%)	0.157	13 (12.15 %)	27 (26.73%)	0.008
Sugars (g)	23 (21.50%)	21 (20.19%)	0.706	22 (20.56%)	21 (21.00%)	0.827	21 (19.63 %)	28 (27.72%)	0.117

Value in parenthesis denote percentages of children.
p-values from McNemar test between Day1 and Day 90 for various groups
